# Books and Pamphlets Received

**Published:** 1886-12-20

**Authors:** 


### BOOKS AND PAMPHLETS RECEIVED.

The *Magazine of American History* closes its sixteenth volume with an exceptionally bright and readable December number. The vigor and grace with which this periodical moves forward in its well regulaed and prosperous course, inspiring fresh love of country with every monthly issue, is a source of pride to the American people. The frontispiece this month is an admirable portrait of Major-General Halleck, made expressly for this magazine from the painting in possession of General George W. Cullum ; it accompanies a paper of surpassing interest to all classes of readers, entitled "Misunderstandings; Halleck and Grant," by General James B. Fry. "The Swamp Angel," the name given to the gun which in 1863 was used in firing on Charleston, is the title to a notable paper by William S. Stryker, adjutant-generel of New Jersey, illustrated with portraits of the officers on that occasion, and other pictures; and General Lec concludes his interesting series "From Cedar Mountain to Chantilly." John Gilmary Shea, LL. D., contributes a short paper on "Beaujeu and Fort Du Quesne;" and Hon. Horatio King writes of "Lincoln and McClellan." The departments are crowded with choice entertainment. This magazine is giving to authentic history the life, animation, interest and intensity it has long needed. $5.00 a year in advance. Published at 30 Lafayette Place, New York City.

*Outline of the Pathology and Treatment of Syphilis and Allied Veneral Diseases.* By Hermann Von Zeissl, M. D., late Professor at University of Vienna. Seeond edition, revised by Maximilian Von Zeissl, M. D., Private Docent for diseases of the skin and syphilis at the Imperial Royal University of Vienna. Authorized edition; translated with notes by H. Raphel, M. D., Attending Physician for diseases of the Genito-Urinary Organs and Syphilis, Bellevue Hospital, out-patient department, etc. New York.: D. Appleton & Co. 1886.

The above work of 402 pages is "a treatise on syphilis by one who has devoted his entire life to the study of this disease, and whose experience is the result of observation and treatment of upward of thirty thousand patients, in private practice and in the wards of the Allgemeine Krankenhaus of Vienna." It is regarded as a work of great research and ability, and eminently practical.

Of the deaths, there occurred 14 from exhausted mania, 13 from epilepsy, 7 from marasmus, 6 from consumption, 4 from apoplexy, 5 from old age, 3 from dropsy, 3 from heart disease, 3 from paralysis, 2 from convulsions, 2 from diarrhœa, 1 from cancer, 1 from inflammation of the stomach, 1 from anæmia, 1

from nervous exhaustion, 1 from erysipelas, 1 from congestion of the kidneys, 1 from chronic exhaustion.

There were, also, colored male lunatics, 126; colored male epileptics, 30; colored male idiots, 10—total colored males, 166; colored female lunatics, 170; colored female epileptics, 12; colored female idiots, 2—total colored females, 184—total colored, 350.

Of the deaths, there occurred from exhausted mania 13, consumption 8, apoplexy 4, epilepsy 5, paralysis 3, softening of brain 2, marasmus 2, chronic diarrhœa 2, heart disease 2, old age 2, dropsy 1, pneumonia 1, diarrhœa 1, convulsions 1, scrofula 1, general dropsy 1, congestion of the brain 1, continued fever 1.

---

*Transactions of the Medical Association of Georgia*, Thirty-seventh Annual Session, held at Augusta, April, 1886.

The book is of somewhat larger size than usual, containing 376 large octavo pages; the binding is neat and beautiful, but it would seem unnecessarily expensive, especially as we are therein told by the Chairman of the Publishing Committee that a number of papers are omitted and the size of the book is limited by deficiency of funds in the hands of the Treasurer. The paper of Dr. Theo. Lamb, of Augusta, is omitted because it was published in a medical journal, and yet the address of Dr. Nunn is included in the book though it also was published in the Atlanta Medical Journal. Dr. Logan's paper was also published in the Atlanta Medical Journal but does not appear in the book of Transactions, for the reason, we are told, it was not furnished the committee; yet it was read and discussed in the Association! It would seem, and has been so understood, that all papers read before the body become the property of the Association, and that the President's address should be subject to the same rule, as to publication in a medical journal, that pertains to other papers. The fact that a number of papers have been omitted, it strikes us, must prove rather unpleasant to the medical brethren who are the authors of them, and the discrimination which enabled the committee to decide upon those which were entitled to the honor of publication must have been exceedingly delicate and embarrassing. We have not space in the present issue for comment upon several valuable papers which the book contains.

*Physician's Visiting List*, 1887. (Lindsay & Blakiston's.) Now ready. Thirty-sixth year of its publication.

Contents: Superior Lead Pencil; Calendar for 1887 and 1888; Marshall Hall's Ready Method in Asphyxia; Poisons and Antidotes; a Description of the Metric or French Decimal System of Weights and Measures; Dose Table; Disinfectants and Disinfecting; Examination of Urine, prepared by Dr. Judson Daland, based upon Prof. James Tyson's "Handbook for Practical Examination of Urine"; List of Standard Reference Books; Incompatibles; Table for Calculating the Period of Utero-Gestation; List of New Remedies; Sylvester's Method for Artificial Respiration, illustrated; Diagram of the Chest.

NEW FEATURES.—A glance at the above contents will show several nes and practical features that have been suggested by our experience, or by those who have used the Visiting List for many years. These additions have been made, however, without increasing the size of the book and without altering its character or general arrangement.

SIZES AND PRICES.—Regu.ar edition: For 25 patients weekly, $1; for 50 patients, $1.25, 75 patients, $1.50; 100 patients, $2; 50 patients weekly, 2 vols. (January to June--July to December), $2.50; 100 patients, 2 vols. (January to June—July to December), $3. Interleaved edition: For 25 patients weekly, $1.25; for 50 patients, $1.50; 50 patients, 2 vols., $3. Perpetual edition, without dates, can be commenced at any time and used until full: For 25 patients, interleaved, $1.25; for 50 patients, $1.50.

This Visiting List can be bought through any bookseller, or, upon receipt of the price, we will send it, postpaid, to any address. Send for our complete Catalogue of Medical Books.

P. BLAKISTON, SON & CO.,
1012 Walnut street, Philadelphia, Pa.

